# FDA approval of mitomycin hydrogel (Zusduri): a new non-surgical era for non-muscle-invasive bladder cancer (NMIBC)

**DOI:** 10.1097/MS9.0000000000005163

**Published:** 2026-06-09

**Authors:** Shiza Abid, Nikhla Qasim, Ahmad Qaisar, Abdullah Nadeem, Javeria Saeed, Syed Usman Ali Shah, Amrah Abdul Samad, Tagwa Kalool Fadlalla Ahmed

**Affiliations:** a Department of Medicine, Ayub Medical College, Abbottabad, Pakistan; b Department of Medicine, Fatima Memorial Hospital College of Medicine and Dentistry, Lahore, Pakistan; c Department of Medicine, Dow Medical College, Karachi, Pakistan; d Department of Medicine, Afhad University for Women, Omdurman, Sudan

**Keywords:** bladder cancer, mitomycin, Zusduri

## Abstract

Non-muscle-invasive bladder cancer (NMIBC) continues to present substantial management challenges, largely due to its high likelihood of recurrence and the routine need for repeated surgical procedures. Standard approaches, including transurethral resection of bladder tumor (TURBT) followed by intravesical therapies such as Bacillus Calmette Guerin or chemotherapy, are effective for many patients but are often limited by toxicity, procedural burden, and variable durability of response. In this context, the recent Food and Drug Administration approval of Zusduri, an intravesical mitomycin hydrogel, represents a noteworthy advancement for adults with recurrent low-grade, intermediate-risk NMIBC. This formulation is designed to transition into a gel within the bladder, extending the drug’s urothelial contact time and enabling in-clinic administration without anesthesia. Results from key clinical trials, i.e., OPTIMA II and ENVISION, reported meaningful chemoablative activity, achieving complete response rates, with a sizable proportion of responders remaining disease-free after 1 year. Most adverse events were manageable and localized, although isolated severe effects were documented. Despite this progress, questions regarding long-term recurrence patterns, broader accessibility, and cost remain unanswered and require future investigation. As real-world data accumulate, mitomycin may emerge as an important addition to the therapeutic landscape, offering a non-surgical option that could lessen dependence on repeated operative interventions and improve convenience for patients with NMIBC.

## Introduction

Bladder cancer is a biologically heterogeneous disease encompassing non–muscle-invasive bladder cancer (NMIBC) and muscle-invasive bladder cancer (MIBC), each with distinct clinical behavior and outcomes. NMIBC accounts for nearly 75% of all newly diagnosed bladder cancer cases in the United States[1]. NMIBC is defined by disease confined to the urothelial mucosa and submucosa and includes papillary non-invasive tumors (Ta), tumors invading the lamina propria (T1), and flat high-grade lesions known as carcinoma *in situ* (CIS)[1]. Urothelial carcinoma is the predominant histologic subtype across these categories. Bladder cancer exhibits a marked demographic disparity, occurring approximately three times more often in men than women. It is the fourth most common solid malignancy among men, with an estimated 16 700 deaths in 2023, disproportionately affecting men and older adults[2]. The incidence is highest in white individuals compared with other racial groups[2]. Tobacco exposure, particularly cigarette smoking, remains the single most important modifiable risk factor for bladder cancer. The pathogenesis involves absorption of carcinogens into the bloodstream, which are filtered by the kidneys and concentrated in urine, leading to prolonged exposure of the urothelial lining to DNA-damaging agents[3]. Additional risk factors include occupational exposure to aromatic amines (paints, dyes, and petroleum products), prior pelvic radiation, use of chemotherapeutic agents such as cyclophosphamide, and genetic alterations involving HRAS, RB1, PTEN/MMAC1, NAT2, and GSTM1[3]. Clinical presentation varies, with painless hematuria being the most common initial symptom. Patients may also experience dysuria, urinary frequency, or urgency. In advanced cases, obstructive symptoms, flank pain, abdominal discomfort, or bone pain may occur, significantly affecting quality of life[3]. Diagnosis relies on cystoscopy, which remains the gold standard for detecting and monitoring NMIBC[4]. Urine cytology serves as a widely used non-invasive test, though its sensitivity is limited, particularly for low-grade tumors. CIS lesions may be difficult to visualize and sometimes require mapping biopsies for detection[4]. Although NMIBC is often diagnosed early, it can cause significant physical and psychological burden, with risks of recurrence and progression that underscore the urgent need for effective and well-tolerated treatments. Our article adheres to the TITAN 2025 guidelines for transparency and integrity in academic publishing[5].

## Current treatment modalities

The gold-standard first-line procedure in NMIBC diagnosis and management is transurethral resection of bladder tumor (TURBT), as strongly recommended by the European Association of Urology (EAU) guidelines for its diagnostic, prognostic, and therapeutic roles[6]. The guidelines stress the importance of complete resection with inclusion of detrusor muscle (muscularis propria) to minimize understaging, especially to exclude T2 disease[6]. TURBT not only removes visible tumors but also supplies tissue for accurate histopathological assessment of grade, stage, and other prognostic features[6]. Despite its central role, patients with high-risk NMIBC remain at substantial risk of recurrence and progression. Based on European Organization for Research and Treatment of Cancer-derived risk estimates (as used in the EAU risk tables), 10-year progression in these patients is 14% (CI: 11–18%)[6].

In contrast, intravesical chemotherapy reduces post-TURBT recurrence; one meta-analysis showed markedly lower intravesical recurrence with a single postoperative instillation (epirubicin or mitomycin-C) versus TURBT alone[7]. Other randomized and comparative studies support gemcitabine and epirubicin as immediate instillations and as options in selected Bacillus Calmette Guerin (BCG) -unresponsive or recurrent NMIBC[8]. However, local toxicities, including chemical cystitis, are frequent; moreover, intravesical therapy is contraindicated when bladder perforation is suspected due to the potential for drug extravasation[8].

Intravesical BCG is an established immunotherapeutic approach. A population-based, long-term cohort demonstrated that BCG therapy in high-risk NMIBC was associated with substantial reductions in both recurrence and progression over 15 years[9]. Comparative evidence from a large network meta-analysis shows that standard-dose BCG provides strong efficacy in reducing recurrence compared to intravesical chemotherapies, although differences in progression were less clear in some comparisons[10]. However, this efficacy comes at the cost of increased overall treatment-related toxicity: the analysis found a significantly higher risk of adverse events with standard-dose BCG (vs. lower-dose), though it does not always distinguish between local urinary symptoms (such as cystitis or dysuria) and rare systemic BCG-related complications[10].

Despite strong efficacy, local and systemic side effects and contraindications of current treatments necessitate alternatives with comparable benefit and better tolerability. In this context, emerging molecular targets are gaining attention. Recent preclinical studies have identified NADSYN1 as a potential oncogenic driver in bladder cancer, with its expression modulated by prohibitin[11]. Targeting this pathway has demonstrated suppression of tumor growth and metastasis in experimental models, underscoring its potential as a novel therapeutic target. However, clinical validation remains essential before translation into routine practice[11].

Mitomycin C (MMC) shows promise in this regard; in a large meta-analysis, single-dose and short-maintenance MMC significantly reduced recurrence versus TURBT alone, supporting its role in low- and intermediate-risk NMIBC[12]. Recurrence and progression outcomes with MMC were comparable to those of other agents, including gemcitabine and docetaxel, while exhibiting a generally favorable safety profile; collectively, these findings position mitomycin as a promising therapeutic option in contemporary intravesical management[12].

## Iqirvo Zusduri (mitomycin), the first non-surgical breakthrough in NMIBC treatment

**Figure 1.**
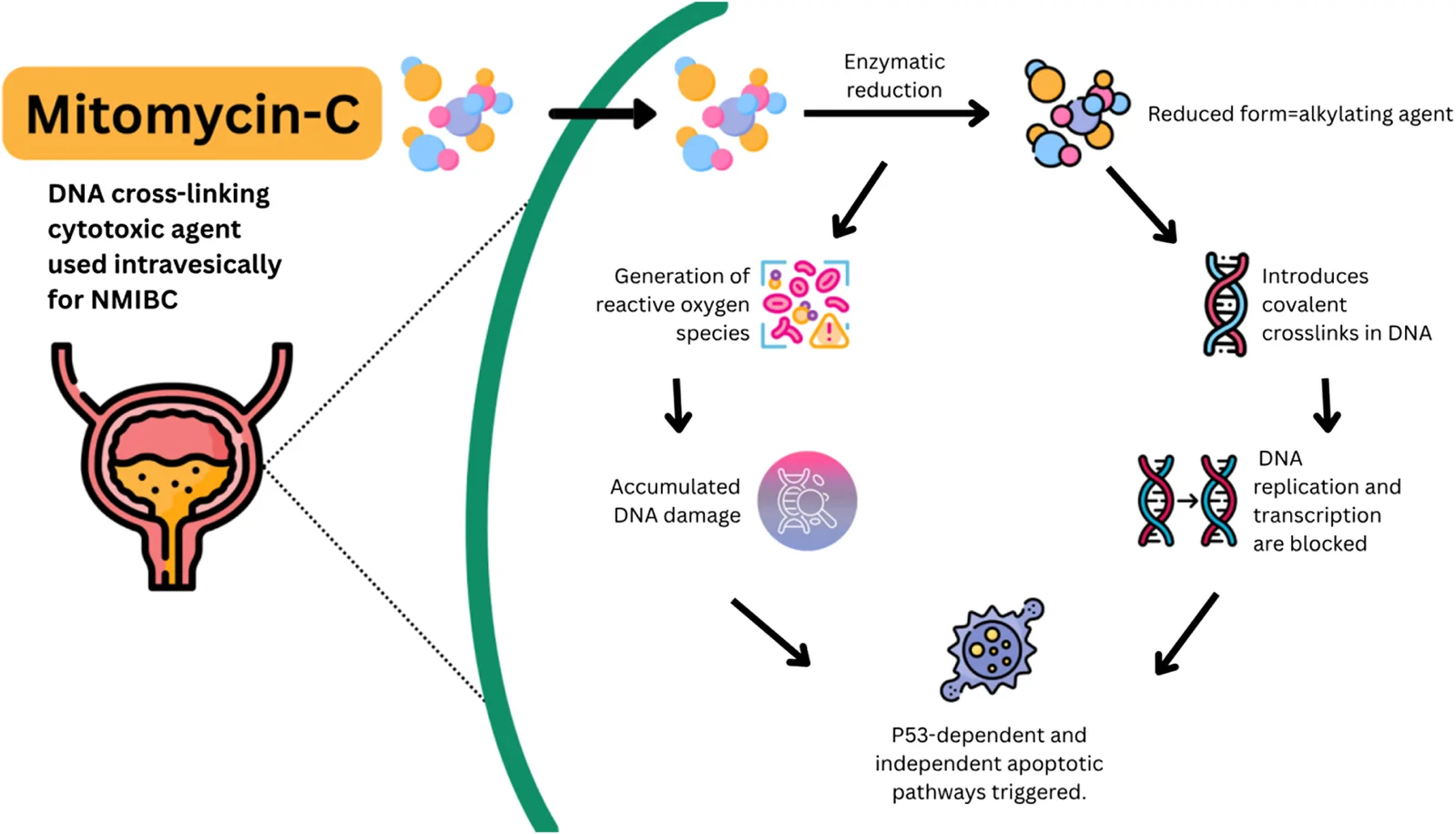
Schematic representation of mitomycin’s mechanism of action.

On 12 June 2025, a major leap forward in NMIBC care took place with the Food and Drug Administration (FDA) approval of mitomycin intravesical solution under the trade name Zusduri. It marks the first non-surgical development for adult patients facing recurrent low-grade intermediate-risk bladder cancer. The prevailing standard of care for NMIBC relies on enduring repetitive surgical procedures named TURBT, performed usually under general anesthesia[13]. The revolutionary approach of Zusduri lies in its intravesical administration as a non-surgical, outpatient procedure that does not require anesthesia or inpatient admission. In comparison with TURBT, which is associated with a high tumor recurrence rate, Zusduri has demonstrated considerable potential in significantly reducing repeat surgical interventions. Mitomycin is a chemotherapeutic alkylating agent that gets activated within tumor cells, forming reactive species that cross-link the cell DNA, thus inhibiting DNA replication and initiating tumor cell apoptosis[14]. Figure 1 shows a schematic representation of its mechanism of action. Additionally, its exclusive hydrogel-based formulation allows prolonged contact of mitomycin infusion with the bladder urothelium, thus enhancing local drug absorption and improving tumor ablation efficacy[15]. Mitomycin has a notable long-term history of use as an intravenous chemotherapeutic regimen in several malignancies, including breast and lung cancer. Its cytotoxic action against proliferating cells also provides benefits against stomach and pancreatic cancers[14].

## Evidence from clinical trials

The FDA approval of Zusduri was based on the robust outcomes of the ENVISION and OPTIMA II trials, which aimed to evaluate the efficacy and safety of mitomycin. The OPTIMA II is a single-arm phase 2 trial that enrolled 63 biopsy-proven LG-IR NMIBC patients. Six once-weekly intravesical mitomycin instillations were administered to the patients. The trial demonstrated a complete response rate (CR) of 65%, and among those with CR, 61% remained disease free at 12 months post-initiation of treatment[16]. The ENVISION trial is a phase 3 single-arm study that recruited 240 patients with biopsy-confirmed recurrent low-grade, intermediate-risk, non-muscle invasive bladder cancer (LG-IR NMIBC). Out of the 240 patients enrolled, 228 received once-weekly mitomycin intravesical instillation for 6 weeks[15]. The CR at 3 months after the start of the treatment was taken as the primary endpoint of the study, where a complete response meant the absence of visible tumor on cystoscopy, negative urine cytology, and negative biopsy. The secondary key endpoints in the ENVISION trial were the durability of complete response, duration of response, safety, and tolerability. The trial concluded that the 6-weekly mitomycin primary chemoablation therapy in patients with low-grade, recurrent NMIBC could result in an 80% CR rate, with 82% of these patients experiencing no recurrence for 1 year[15]. The ATLAS trial also assessed the efficacy and safety of mitomycin, but it was terminated early due to FDA concerns[16]. The outcomes and results of these trials are summarized in Table 1.

**Table 1:** Key findings of clinical trials.

Trial name (NCT ID)	Years	Population	Primary endpoints	Secondary endpoints	Results
OPTIMA II	2020–2022	63 patients with recurrent IR-LG NMIBC	CR rate at 3 months	Durability at 6, 9, 12 months, safety	CR 65% at 3 months, 95% still disease free at 6 months, 73% at 9 months, 61% at 12 months. TEAEs; mild to moderate.
Phase 2b					
(NCT03558503)					
ATLAS	2023–2024	282 patients with new or recurrent IR-LG NMIBC. (Intervention; mitomycin *n* = 142, Control; TURBT *n* = 140)	CR rate at 3 months, DFS	DOR, safety	CR 64.8% in mitomycin group vs 63.6% in TURBT group, 79.7% DFS at 12 months in intervention vs 67.7% at 12 months in control group
Phase 3					
(NCT04688931)					
ENVISION	2023–2024 (ongoing)	240 patients with recurrent LG-IR NMIBC	CR rate at 3 months, durability in survivals	DOR at 12, 15, 18 months, safety	CR 79.6% at 3 months, 82.3% DOR at 12 months, 80.9% at 15 months and 18 months. Serious adverse events-12%
Phase 3					
(NCT05243550)					

CR, complete response rate; DFS, disease-free survival; IR-LG NMIBC, intermediate risk, low-grade, non-muscle invasive bladder cancer; DOR, duration of response.

## Usage guidelines and administration

Zusduri (mitomycin intravesical solution) has a proven efficacious safety and tolerability profile with minimal side effects. The dosage of Zusduri tested across clinical trials is 56 ml of solution containing 75 mg of mitomycin, supplied as a reverse thermal gel formulation. The only approved route of administration is instillation through a catheter into the bladder cavity, with the administration frequency being once weekly for 6 consecutive weeks^[^17^]^. The significant contraindications are a perforated bladder, a prior allergic reaction to mitomycin, severe renal impairment (eGFR less than 30 ml per minute), and a compromised bladder mucosa. Other precautions, due to its teratogenic potential in animal studies, include contraception for 6 months in women and 3 months in men post-treatment. Moreover, breastfeeding is also discouraged during the treatment and for 1 week after the final dose. Zusduri is primarily metabolized through the liver, with a half-life of 8–48 minutes. However, 10% of the unchanged product is excreted via the kidneys[17].

## Limitations and future prospects

The common side effects include disrupted lab values with increased creatinine, potassium, AST/ALT, and eosinophils, and decreased hemoglobin and lymphocyte count. It is shown to cause urinary tract symptoms like dysuria and hematuria. The serious side effects noted in the trials were urinary retention, urethral stenosis, and one fatal cardiac failure event. Although no study has yet been conducted to evaluate its drug cross-reactivity, caution should still be practiced while prescribing it along with other alkylating agents or nephrotoxic drugs due to its renal excretion^[^18^]^. Even though this approval fulfills an important treatment gap, data on long-term prognosis remain scarce, particularly regarding recurrence rates beyond the observed trial duration. Challenges related to accessibility and cost also persist, as drug availability may differ across regions, and high treatment expenses could limit its use. These considerations demand the need for more real-world studies to better understand the economic burden and ensure equitable access across regions and populations.

## Conclusion

NMIBC continues to pose a significant therapeutic challenge, with high recurrence and progression rates underscoring the need for effective intravesical strategies. Existing treatment options often fall short in providing durable disease control, reinforcing the importance of therapies that can reliably reduce tumor relapse. Mitomycin, with its potent DNA-crosslinking mechanism and established efficacy in lowering postoperative recurrence rates, remains a cornerstone in the contemporary management of NMIBC. Its intravesical administration allows for targeted drug delivery with limited systemic exposure, enhancing tolerability across diverse patient populations. Moreover, the generally manageable safety profile of mitomycin supports its continued role as a practical and impactful treatment option. As the therapeutic landscape evolves, optimizing and integrating proven modalities like mitomycin hold promise in improving outcomes and delivering more durable disease control for patients with NMIBC.

## Data Availability

The data supporting the findings of this study are available from the corresponding author upon reasonable request.

## Research registration unique identifying number (UIN)

This study was not prospectively registered; therefore, a research registration unique identification number is not applicable.
